# Inhibition of SK4 Potassium Channels Suppresses Cell Proliferation, Migration and the Epithelial-Mesenchymal Transition in Triple-Negative Breast Cancer Cells

**DOI:** 10.1371/journal.pone.0154471

**Published:** 2016-04-28

**Authors:** Panshi Zhang, Xiaowei Yang, Qian Yin, Jilin Yi, Wenzhuang Shen, Lu Zhao, Zhi Zhu, Jinwen Liu

**Affiliations:** 1 Department of Thyroid and Breast Surgery, Tongji Hospital, Tongji Medical College, Huazhong University of Science and Technology, Wuhan, China; 2 Department of General Surgery, the First Affiliated Hospital, Anhui Medical University, Hefei, China; University of South Alabama Mitchell Cancer Institute, UNITED STATES

## Abstract

Treatments for triple-negative breast cancer (TNBC) are limited; intermediate-conductance calcium-activated potassium (SK4) channels are closely involved in tumor progression, but little is known about these channels in TNBC. We aimed to investigate whether SK4 channels affect TNBC. First, by immunohistochemistry (IHC) and western blotting (WB), increased SK4 protein expression in breast tumor tissues was detected relative to that in non-tumor breast tissues, but there was no apparent expression difference between various subtypes of breast cancer (*p*>0.05). Next, functional SK4 channels were detected in the TNBC cell line MDA-MB-231 using WB, real-time PCR, immunofluorescence and patch-clamp recording. By employing SK4 specific siRNAs and blockers, including TRAM-34 and clotrimazole, in combination with an MTT assay, a colony-formation assay, flow cytometry and a cell motility assay, we found that the suppression of SK4 channels significantly inhibited cell proliferation and migration and promoted apoptosis in MDA-MB-231 cells (*p<*0.05). Further investigation revealed that treatment with epidermal growth factor (EGF)/basic fibroblast growth factor (bFGF) caused MDA-MB-231 cells to undergo the epithelial-mesenchymal transition (EMT) and to show increased SK4 mRNA expression. In addition, the down-regulation of SK4 expression inhibited the EMT markers Vimentin and Snail1. Collectively, our findings suggest that SK4 channels are expressed in TNBC and are involved in the proliferation, apoptosis, migration and EMT processes of TNBC cells.

## Introduction

Breast cancer is the most frequently diagnosed cancer and the leading cause of cancer death among females worldwide, with an estimated 1.7 million new cases and 521,900 deaths in 2012 [1]. TNBC is one major subtype of breast cancer, defined as tumors that lack expression of the estrogen receptor (ER), the progesterone receptor (PR), and HER2 [2]. TNBC accounts for approximately 15% of breast cancers and is associated with a higher risk of relapse and a worse overall survival compared with other breast cancer subtypes, including ER-positive (luminal) and HER2-positive (HER2 over-expression) subtypes [3]. Although sensitive to chemotherapy, TNBC has relatively limited targeted drug treatment options at present. Thus, it is crucial to further explore the biological features and mechanism of TNBC progression to develop potent clinical targets [4].

Intermediate-conductance calcium-activated potassium channels, abbreviated as SK4, KCa3.1 or hIKCa1, have been recently studied in various types of cancer [5]. Blockage of SK4 inhibits the progression of human endometrial cancer [6]. Our previous research demonstrated that SK4 plays an important role in the proliferation of hepatocellular carcinoma cells [7]. Another study indicated that SK4 can mediate apoptosis in D54-MG glioma cells through the activation of the intrinsic pathway [8]. Moreover, inhibition of SK4 activity reduces cell motility in glioblastoma-derived cancer stem cells [9]. The above studies provide evidence that SK4 channels participate in cancer progression, including cell proliferation, apoptosis and metastasis.

In addition, recent research indicates that SK4 channels participate in the regulation of the EMT. In idiopathic pulmonary fibrosis, SK4 channels promote the differentiation of fibroblasts to myofibroblasts [10]. In colorectal cancer, SK4 participates in EMT induced by regenerating liver-3 [11]. Another study proves that inhibition of SK4 reduces the TGF-β1-induced myofibroblast phenotype transition of mesangial cells [12].

These findings support the increased interest in investigating SK4 as a tumor marker and therapeutic target for cancers. Although SK4 in breast cancer has been studied in detail, little is known regarding SK4 in TNBC. Thus, in this study, we investigated the relative downstream effects of SK4 on TNBC, with a particular focus on changes in cell proliferation, apoptosis and migration. We also investigated whether SK4 channels are involved in the EMT process of TNBC. We found significant expression of SK4 channels in TNBC tissues and cell lines and identified the electrophysiological function of SK4 channels in MDA-MB-231 cells. We also showed that the inhibition of SK4 channels suppressed cell proliferation and migration and the EMT process.

## Materials and Methods

### Ethics Statement

The study considered Declaration of Helsinki to be a statement of ethical principles. Written informed consent was obtained from all subjects, and all protocols were approved by the Ethical Committee of Tongji Hospital, Tongji Medical College, Huazhong University of Science and Technology (IRB ID: TJ-C20120212).

### Cell culture and treatments

Four classic human breast cancer cell lines, MCF-7, T47D, MDA-MB-231 and MDA-MB-468, were obtained from the China Center for Type Culture Collection (Wuhan University, China; Catalogue number: GDC055, GDC045, GDC0297) and Type Culture Collection of the Chinese Academy of Sciences (Shanghai, China; Catalogue number: TCHu136) separately in 2012. MCF-7 cells were cultured in high-glucose Dulbecco’s Modified Eagle’s Medium (DMEM; Gibco, USA) supplemented with 10% heat-inactivated fetal bovine serum (FBS; Gibco, USA). T47D, MDA-MB-231 and MDA-MB-468 cells were cultured in Roswell Park Memorial Institute (RPMI) 1640 medium (Gibco, USA) supplemented with 10% heat-inactivated FBS. All cell lines were maintained at 37°C in a 5% CO_2_ humidified atmosphere. The media were changed every second or third day.

For some experiments, cells were incubated in medium containing either 1-(2-chlorophenyl)diphenylmethyl-1H-pyrazole (TRAM-34; Sigma-Aldrich, USA), clotrimazole (Sigma-Aldrich, USA), transforming growth factor-β1 (TGF-β1; PeproTech, USA), EGF (PeproTech, USA) and bFGF (PeproTech, USA).

### Tissue specimens

Breast cancer tissues were obtained from 50 patients who were admitted to the Department of Thyroid and Breast Surgery, Tongji Hospital, Tongji Medical College, Huazhong University of Science and Technology between 2012 and 2015. All patients received surgery treatment with no chemotherapy, radiotherapy or any other adjuvant therapy. All samples were subsequently diagnosed as primary breast cancers (including a molecular classification) by the Department of Pathology, Tongji Hospital.

### Immunohistochemistry and scoring

Tissues were fixed, paraffin embedded, and serially sectioned at a 4-μm thickness. Then, the sections were stained using the 2-step plus detection system (Golden Bridge International, USA) according to the manufacturer’s instructions. Briefly, the sections were deparaffinized, rehydrated, blocked with H_2_O_2_, washed and then incubated with rabbit polyclonal anti-SK4 antibody (1:200) (Bioss, Beijing, China) overnight at 4°C. The next day, the slides were washed, incubated with Reagent Polymer Helper, washed, incubated with Reagent Polyperoxidase-anti-rabbit IgG and developed with diaminobenzidine (DAB) solution. Then, the slides were examined under an optical microscope (Motic, Xiamen, China).

A specialist histopathologist scored the breast cancer tissues. Briefly, scoring standards included the intensity of staining, scored as 0 (negative), 1 (weak), 2 (moderate), and 3 (strong), and the percentage of positive cells, scored as 0 (no staining), 1 (<25%), 2 (25–50%), 3 (50–75%), and 4 (>75%). The product of the score of the staining intensity and the score of staining extent constituted the total score. Each slide was scored at 5 different fields under high magnification, and the average was obtained. Negative cases were defined as a score of <2; weak positive for a score of 3–6; positive for a score of 6–8; and strong positive for a score of >8. For quantitative analysis, low SK4 expression was defined as a score of ≤6, and high expression was defined as a score of >6.

### Real-time quantitative PCR

Cellular total RNA was extracted using TRIzol Reagent (Invitrogen, USA) according to the manufacturer’s instructions. A ReverTra Ace qPCR RT Kit (TOYOBO, Japan) was used to synthesize cDNA. Real-time PCR was performed on a StepOne Plus Real-Time PCR System (Applied Biosystems, USA) using SYBR Green Real-time PCR Master Mix (TOYOBO, Japan). The housekeeping gene GAPDH was used as an internal control. The primer sequences are shown in S1 Table. All reactions were run in triplicate. The relative amount of target gene was calculated using the formula 2^-ΔΔCt^.

### siRNA knockdown of SK4 expression

A negative control siRNA and 3 siRNA molecules targeting SK4 were designed and synthesized (RiboBio, Guangzhou, China; S2 Table). Transfection was performed according to manufacturer’s instructions: cells were plated in 6-well plates at 1×10^5^ cells per well with complete medium and cultured overnight. Then, each well was transfected with 50 nM siRNA using riboFECT CP Reagent (RiboBio, Guangzhou, China). Western blotting and real-time PCR were used to determine the siRNA efficiency.

### Western blotting

According to the manufacturer’s instructions, proteins were extracted from cells or tissues using Western-IP Lysis Buffer (Beyotime, Shanghai, China). The protein concentration was determined using a bicinchoninic acid (BCA) protein concentration determination assay kit (Beyotime, Shanghai, China). Prepared samples were electrophoresed in a 10% SDS-PAGE gel and blotted onto a polyvinylidene fluoride (PVDF) membrane (Millipore, USA) using the Tetra Handcast system (Bio-Rad, USA). The membranes were blocked for 3 h at room temperature and incubated overnight at 4°C with an appropriate primary antibody in Tris-buffered saline with 0.05% Tween (TBST) containing 5% non-fat milk. After washing in TBST, the membranes were incubated with secondary antibody, washed and visualized using a supersensitive enhanced chemiluminescence (ECL) kit (Beyotime Institute of Biotechnology, China) according to the manufacturer’s protocol. The protein bands were detected and quantified using the Gene Gnome Syngene Bio Imaging System (SYNGENE, UK).

Primary antibodies used for Western blotting included a mouse monoclonal anti-SK4 antibody (1:100; Alomone Labs, Israel), a rabbit polyclonal ER antibody (1:1,000; a gift from Dr. Yibing Hu), a rabbit monoclonal anti-EMT antibody sampler kit (1:1,000; Cell Signaling Technology, USA) and a mouse monoclonal anti-tubulin antibody (1:500; abcam, USA).

### Immunofluorescence analysis

Cells were plated on Lab-Tek Chamber Slides (Thermo Fisher Scientific, USA) in complete media at 37°C with 5% CO_2_. Cells were fixed with 1% paraformaldehyde for 20 min at room temperature, permeabilized with 0.1% Triton X-100, blocked in 10% serum at 37°C for 30 min, and finally incubated with the SK4 antibody (1:20) (Alomone Labs, Israel) at 4°C overnight. Then, the cells were incubated with a matched Alexa Fluor 594-conjugated secondary antibody (1:300; Thermo Fisher Scientific, USA) and DAPI for 30 min in dark at room temperature and visualized and recorded using fluorescence microscopy (Nikon, Japan).

### Electrophysiological recordings

Patch-clamp recordings were performed in the whole-cell configuration using a MultiClamp 700A amplifier. Electrodes were fabricated using a Narishige two-stage puller. Electrodes had a resistance of 3–5 MΩ when filled with recording solution and the seal resistance was greater than 2 GΩ. Before whole-cell recordings, the cells were cultured appropriately at a density of 1×10^4^/ml on 35-mm dishes. The bath solution contained (in mM) 140 NaCl, 5 KCl, 3 CaCl_2_, 1.2 MgSO_4_, 10 glucose and 10 HEPES, pH 7.4. The pipette solution contained (in mM) 135 KCl, 2 MgSO_4_, 2.5 ATP, 0.1 EGTA and 10 HEPES, pH 7.2. Free calcium ions applied in the pipette solution were buffered with 10 mM EGTA and calculated using Winmaxc32 software (Chris Patton, Hopkins Marine Station, Stanford University). For example, to produce 350 nM of free calcium, 7 mM CaCl_2_ and 10 mM EGTA were used (pH 7.2). The currents were evoked by step voltage ranging from -100 mV to +100 mV in steps of 10 mV every 100 ms. All data were analyzed by Clampfit, Pulsefit and SigmaPlot software.

### MTT proliferation assay

Cells were plated onto 96-well plates at 5×10^3^ (MDA-MB-231) or 1×10^4^ (T47D) cells per well and divided into control and treatment groups. After 24 h, for the treatment group, TRAM-34 or clotrimazole was added, with concentrations ranging from 4 μM to 20 μM. After an additional 48 h, 20 μl of MTT solution (5 mg/ml in PBS; Biosharp, Hefei, China) was added to each well and incubated (4 h, 37°C). The medium was then removed, and 150 μl of dimethyl sulfoxide (DMSO) was added and incubated (37°C, 10 min). Afterwards, the optical density at 490 nm of each well was measured using a Synergy 2 Muti-mode microplate reader (BioTek, USA). The experiments were performed in quintuplicate and repeated 3 times.

### Apoptosis assay

Cell apoptosis was analyzed using the Annexin V-FITC Apoptosis with propidium iodide (PI) Detection Kit (PeproTech, USA) according to the manufacturer’s instructions. Cells were harvested, quantified, washed in binding buffer and centrifuged. Then, the supernatant was aspirated, and the cells were resuspended in 100 μl of binding buffer per 10^5^ cells. After that, 5 μl of Annexin V-FITC and 10 μl of PI solution per 10^6^ cells was added, mixed well and incubated for 15 min in the dark at room temperature. Then, 400 μl of binding buffer was added, mixed and analyzed using flow cytometry (BD, USA) and FlowJo software. The experiments were repeated 3 times.

### Colony-formation assay

About 200–500 cells per well were seeded in 6-well plates, and 24 h later, 10 μM or 20 μM TRAM-34 was administered. Media were changed twice a week. After approximately 2–3 weeks, colonies were fixed in 1% paraformaldehyde and stained with 0.1% crystal violet. Colonies with no less than 50 cells per colony were counted. The experiments were repeated 3 times.

### Cell motility assay

Four groups were used in the cell motility assay, namely, the parental MDA-MB-231 cells as a control, the cells transfected with SK4 or control siRNA, and the cells treated with 10 μM TRAM-34. Up to 1×10^4^ cells per well were plated in medium containing 5% FBS in the upper chamber of a 24-well Boyden chamber with an 8-μm pore (Corning, USA). For the SK4 siRNA and control siRNA groups, the cells were transfected properly and then plated in the upper chamber at the appropriate time. For the TRAM-34-treatment group, 10 μM TRAM-34 was added to the upper chamber. The lower chamber that served as a chemoattractant was filled with medium containing 15% FBS. Cells were incubated for 24 h at 37°C and 5% CO_2._ Then, non-invading cells were removed with a cotton swab, and cells that had moved through the pores to the lower surface of the membranes were fixed with methanol and stained with 0.1% crystal violet. Five visual fields were randomly selected from each membrane, and the cell numbers were counted via a light microscope. The experiments were performed in triplicate.

### Wound-healing assay

Up to 1×10^6^ MDA-MB-231 cells per well were plated in 6-well plates. Cells were incubated overnight, and 3 parallel wounds per well with the same interval were created using a pipette tip. Images were captured at 0, 12 and 24 h after wounding. The distance migrated by the cells to close the wounded area during the time was measured using ImageJ software. The results are expressed as the wound-healed rate (%), that is, the distance migrated by cells at certain time divided by the wound distance at 0 h. Experiments were performed in duplicate and repeated at 3 times.

### Statistical analyses

Data are expressed as the mean ± standard error (SD). The comparison of SK4 expression in breast cancer tissues was analyzed using Fisher’s exact test. For the other comparisons, Student’s *t*-test or an ANOVA test was used. All statistical evaluation was performed using GraphPad Prism^®^ 5.0. The results were considered significant at *p<0.05, **p<0.01 and ***p<0.001.

## Results

### SK4 proteins are expressed in human primary breast cancer tissue

**Fig 1:**
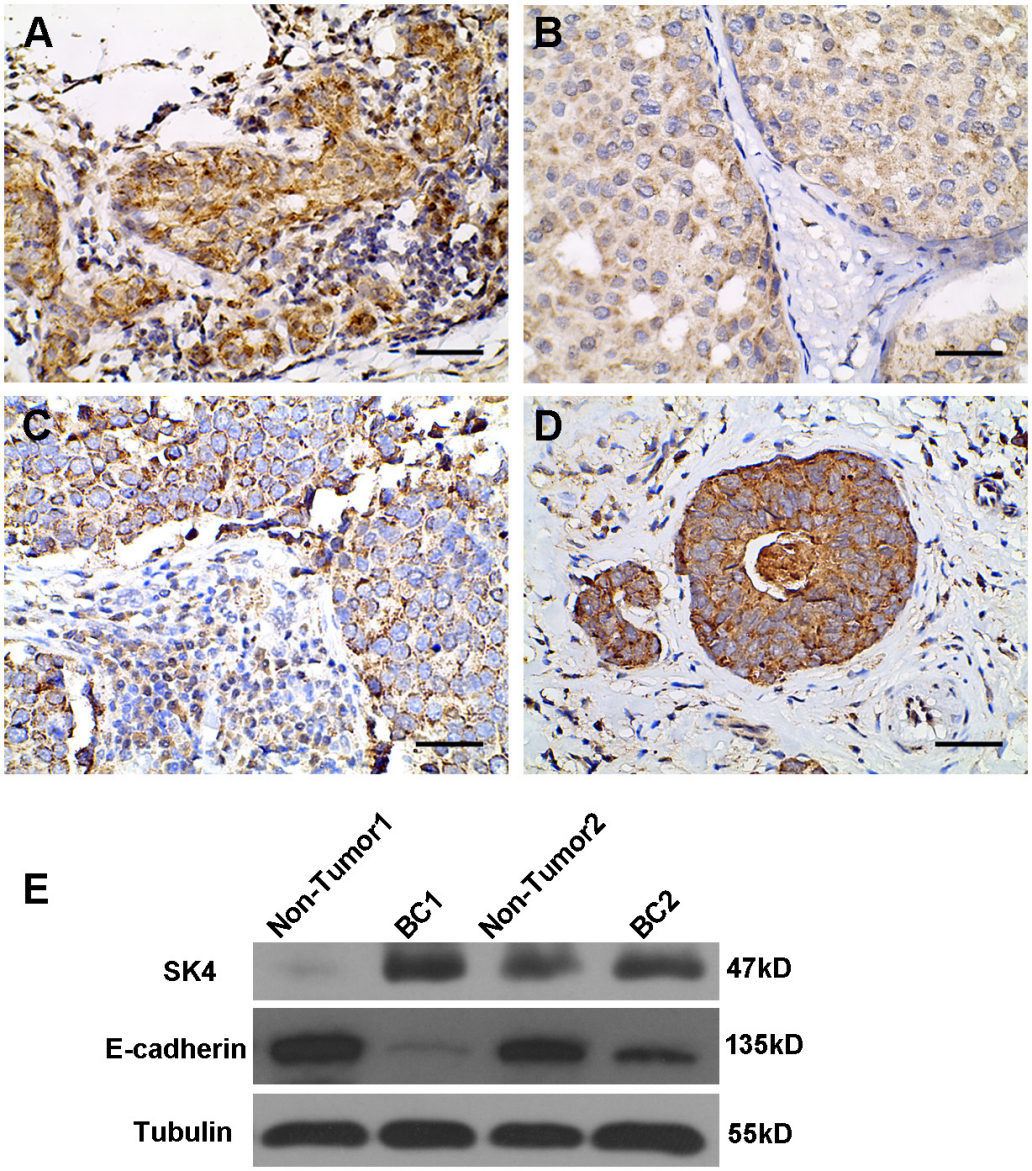
SK4 proteins expressed in breast cancer tissue. (A-D) SK4 IHC in fours subtypes of breast cancer tissues including Luminal A (A), Luminal B (B), HER2 (C), and TNBC (D). Scale bars, 50 μm. (E) Immunoblotting of SK4 and E-cadherin in breast cancer tissues (BC1 and BC2) and non-tumor breast tissues (Non-Tumor1 and Non-Tumor2).

SK4 proteins were first detected by performing IHC on 50 breast cancer samples of various subtypes. In total, there were 12 cases of TNBC, 9 cases of HER2 (ER/PR-negative, HER2-positive) and 29 cases of luminal (ER-positive) breast cancer. The SK4 protein was detectable in all of the studied cases (Fig 1A–1D). Next, we investigated whether SK4 expression correlated with the molecular subtype of breast cancer. We assigned the subtypes into two groups, 38 cases of non-TNBC (luminal and HER2) and 12 cases of TNBC, and used Fisher’s exact test to make a quantitative analysis as reported in Table 1. We found a high SK4 expression in 91.7% (n = 11) and 89.5% (n = 34) of TNBC and non-TNBC cells, respectively, with no significant difference between the two groups (*p*>0.05). We also compared SK4 and E-cadherin protein expression in breast cancer tissues and non-tumor breast tissues using WB (Fig 1E). We observed increased SK4 expression and decreased E-cadherin expression in the tumor tissues.

**Table 1:** Comparison of SK4 expression in tumor subtypes for 50 patients using Fisher’s exact test.

Tumor	SK4-Low	SK4-High	n	*p*
TNBC	1	11	12	>0.05
Non-TNBC	4	34	38	

For the 12 cases of TNBC and 38 cases of luminal and HER2 breast cancer (Non-TNBC), low SK4 expression was defined as an IHC score of ≤6, and high, >6.

### SK4 channels are expressed functionally in breast cancer cell lines

**Fig 2:**
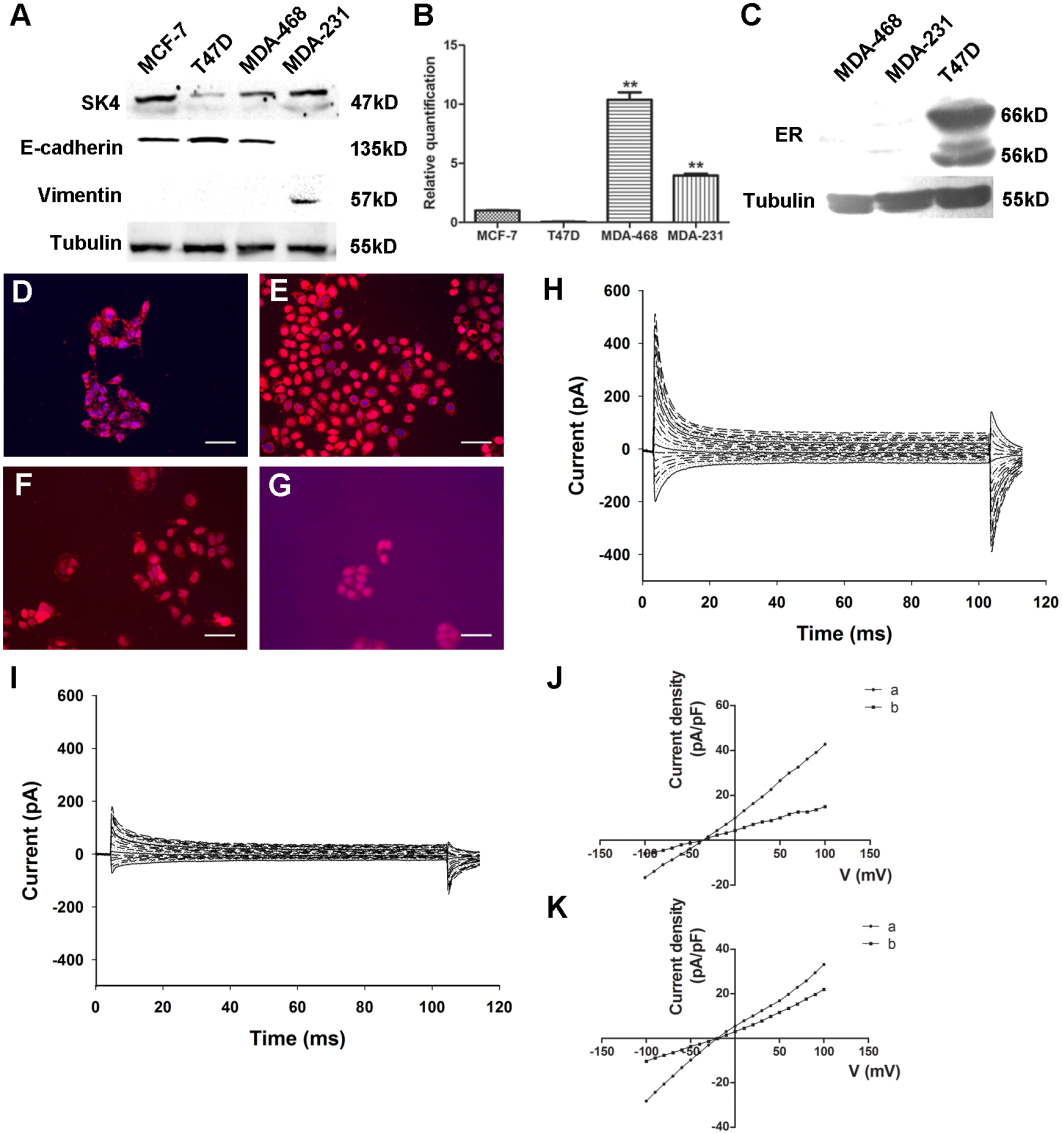
Functional expression of SK4 channels in breast cancer cells. (A) Immunoblotting of SK4 and EMT-related proteins (E-cadherin and Vimentin) in breast cancer cell lines. (B) Comparison of SK4 mRNA expression in 4 breast cancer cell lines as determined by real-time PCR; n = 3. (C) Immunoblotting of ER protein in MDA-MB-468, MDA-MB-231 and T47D cells. (D-G) Immunostaining of SK4 (red) and nuclear marker DAPI (blue) in MDA-MB-231 (D), MDA-MB-468 (E), MCF-7 (F) and T47D (G) cells. Scale bars, 50 μm. (H, I) Whole-cell recording of MDA-MB-231 cells before (H) and after (I) 5-μM TRAM-34 treatment. (J, K) With (J) or without (K) 350 nM free Ca^2+^ in the pipette solution, the voltage-current density curves show the currents changes before (a) and after (b) TRAM-34 treatment. The currents were evoked by step voltage ranging from -100 mV to +100 mV in steps of 10 mV every 100 ms. Dunnett’s Multiple Comparison Test was applied in comparison, **p<0.01.

Four well-characterized breast cancer cell lines, MCF-7, T47D, MDA-MB-231 (MDA-231) and MDA-MB-468 (MDA-468), were included. We first found that SK4 proteins were expressed in MCF-7, MDA-468 and MDA-231 cells but showed little expression in T47D cells (Fig 2A). At the same time, we detected the EMT state of the cell lines and found that MCF-7, T47D and MDA-468 cells expressed the epithelial cell marker E-cadherin but did not express the mesenchymal cell marker Vimentin, whereas MDA-231 cells exhibited the opposite expression pattern (Fig 2A). Next, we measured the expression levels of SK4 mRNA in breast cancer cells. SK4 mRNA was detected in all cell lines (Fig 2B). With MCF-7 cells as the control, the relative SK4 mRNA levels were 1.00 ± 0.06 times in MCF-7 cells, 0.06 ± 0.01 in T47D cells, 10.38 ± 1.09 in MDA-468 cells, and 3.97 ± 0.27 in MDA-231 cells. Again, we investigated the expression of ER in the T47D, MDA-231 and MDA-468 cell lines. Fig 2C shows a clear and specific band in T47D cells but not in MDA-468 or MDA-231 cells, indicating that ER protein was only expressed in T47D cells. We also located the SK4 proteins in these four cell lines. In Fig 2D–2F, SK4 proteins (red color) were clearly detected in these 3 cell lines with an enriched signal on the cell membrane, while the signal was relatively negative in T47D cells (Fig 2G). These data suggested that SK4 protein was expressed in MCF-7, MDA-468 and MDA-231 cells but showed little expression in T47D cells.

Next, we determined whether SK4 channels were functional in MDA-231 cells. First, with 350 nM free Ca^2+^ in the pipette solution and a step voltage stimulation, apparent outward currents were detected (Fig 2H). Three minutes after MDA-231 cells were treated with 5 μM of the SK4-specific blocker TRAM-34, the outward currents decreased apparently (Fig 2I). The voltage-current density curve shown in Fig 2J indicates that TRAM-34 could block the outward currents significantly with no reversal potential change (about -50 mV). When no free Ca^2+^ was added in the pipette solution, the reversal potential turned right to nearly -20 mV, and the block effect of TRAM-34 weakened (Fig 2K).

### The inhibitory effect of the SK4-specific blocker on MDA-MB-231 cell proliferation

**Fig 3:**
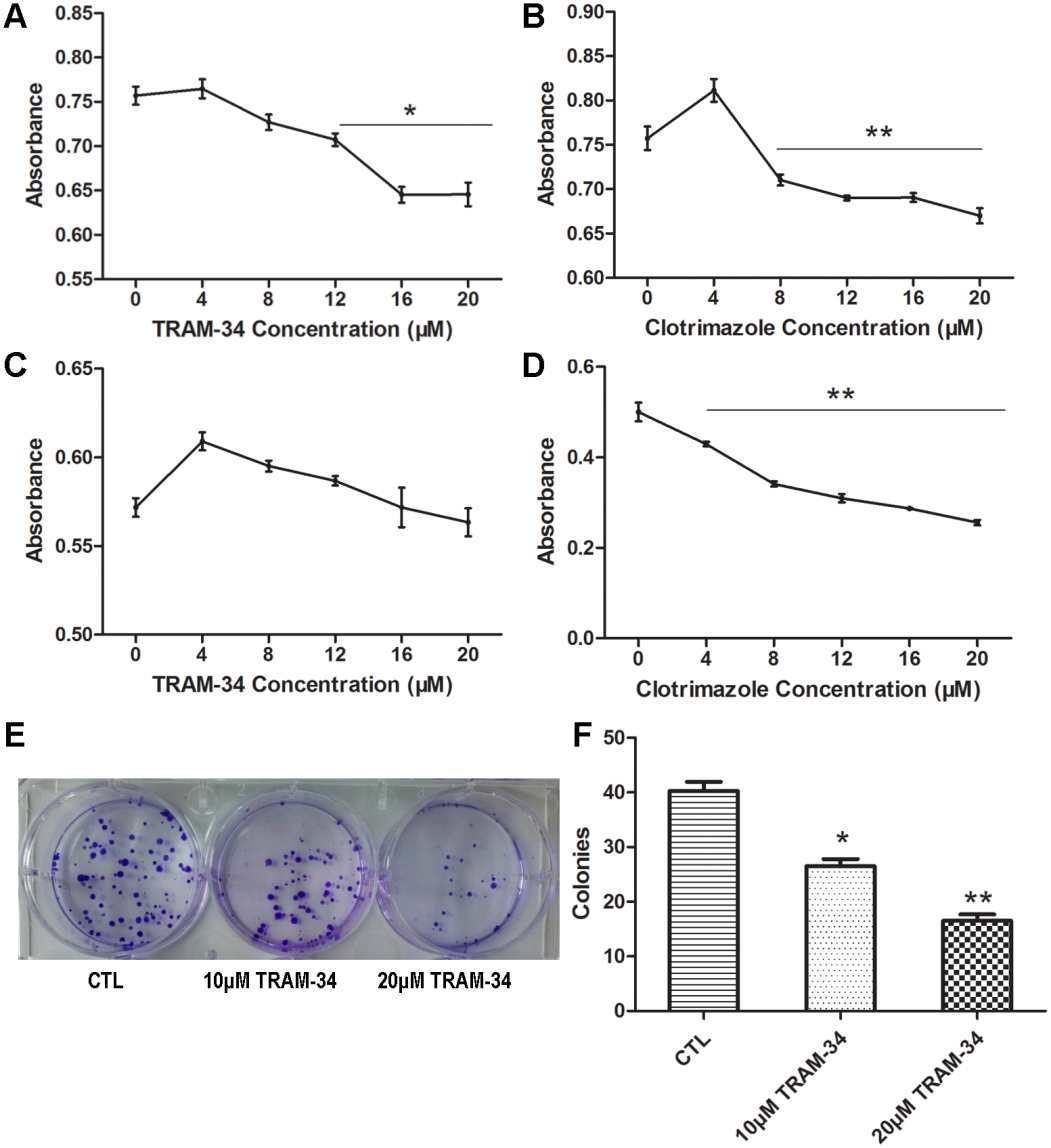
Blockage of SK4 channels inhibits MDA-MB-231 cell proliferation and colony formation ability, but not that of T47D cells. (A-D) Cell growth of MDA-MB-231 (A, B) and T47D cells (C, D) was analyzed using an MTT assay. The two cell lines were treated with 0–20 μM TRAM-34 (A, C) or clotrimazole (B, D) for 48 h, and the absorbance was measured; n = 5. (E, F) Images of the formed MDA-MB-231 colonies in the control group (CTL) and treatment groups (10 μM TRAM-34 and 20 μM TRAM-34); the bar represents separate counts of the colonies; n = 4. The data are presented as the mean ± SD, and Dunnett’s Multiple Comparison Test was applied in comparison. *p<0.05, **p<0.01.

To determine whether SK4 channels have an effect on the proliferation of breast cancer cells, we applied the SK4-specific inhibitors TRAM-34 and clotrimazole to MDA-231 and T47D cells. For the MDA-231 cells, the MTT assay indicated that the absorbance (OD) of the 20-μM TRAM-34- and clotrimazole-treated groups was 0.65 ± 0.02 and 0.67 ± 0.01, respectively, which was significantly lower compared with that of the control group (0.76 ± 0.02; *p<0.05; Fig 3A and 3B). However, for T47D cells, the OD values of the 20-μM TRAM-34 and control group were not apparently different, with values of 0.57 ± 0.01 and 0.56 ± 0.01, respectively (*p>*0.05; Fig 3C). However, the data suggested that clotrimazole (4–20 μM) could inhibit T47D cell proliferation significantly in a dose-dependent manner (**p<0.01; Fig 3D). As a result, TRAM-34 and clotrimazole could inhibit MDA-231 cell proliferation, while T47D cell proliferation could only be inhibited by clotrimazole.

### TRAM-34 suppresses the colony formation of MDA-MB-231 cells

A colony-formation assay was applied to evaluate the effect of TRAM-34 on the colony-formation ability of MDA-MB-231 cells. There were 40 ± 3.4 colonies in the control group, 27 ± 2.6 in the 10-μM TRAM-34-treated group (*p<0.05) and 17 ± 2.4 in the 20-μM TRAM-34-treated group (**p<0.01; Fig 3E and 3F). The colony numbers in the intervention groups were notably reduced compared with the control group, indicating that TRAM-34 could inhibit the colony-formation ability of MDA-231 cells.

### Blockage of SK4 channels promotes MDA-MB-231 cell apoptosis

**Fig 4:**
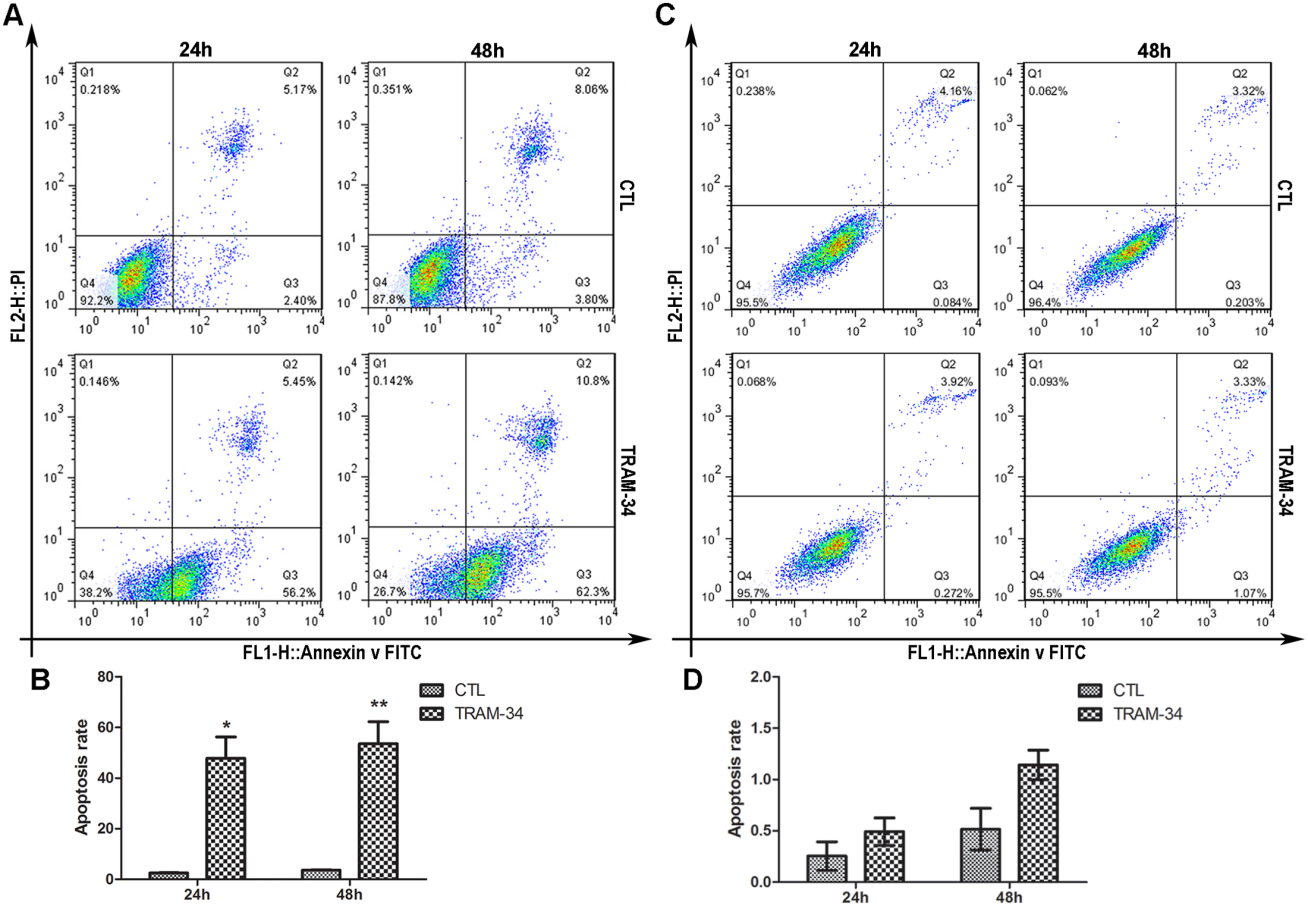
Blockage of SK4 channels promotes apoptosis in MDA-MB-231 cells but not T47D cells. MDA-MB-231 (A, B) and T47D (C, D) cells were treated with 20 μM TRAM-34 for 24 or 48 h, and cell apoptosis was analyzed by Annexin V-FITC/ PI-PE staining and flow cytometry. The bar of MDA-MB-231 indicates that the apoptosis rate of the TRAM-34-treated group increased apparently compared with that of the control (CTL). For T47D, the difference was not significant. The data are presented as the mean ± SD, and unpaired *t* test was applied in comparison. n = 3; *p<0.05, **p<0.01.

We then examined whether TRAM-34 could affect the apoptosis of MDA-MB-231 and T47D cells. For MDA-231, 24 h after treatment with 20 μM TRAM-34, the cell early apoptosis rate (Q3) was 56.2%, and the rate was elevated to 62.3% 48 h later, and both of these rates were apparently increased compared with those of the controls (Fig 4A and 4B), while T47D cells showed no significantly different apoptosis rate between the TRAM-34 group and the control (Fig 4C and 4D). This result indicated that TRAM-34 could facilitate the apoptosis of MDA-MB-231 cells but not T47D cells.

### Down-regulation of SK4 channels inhibits MDA-MB-231 cell migration

**Fig 5:**
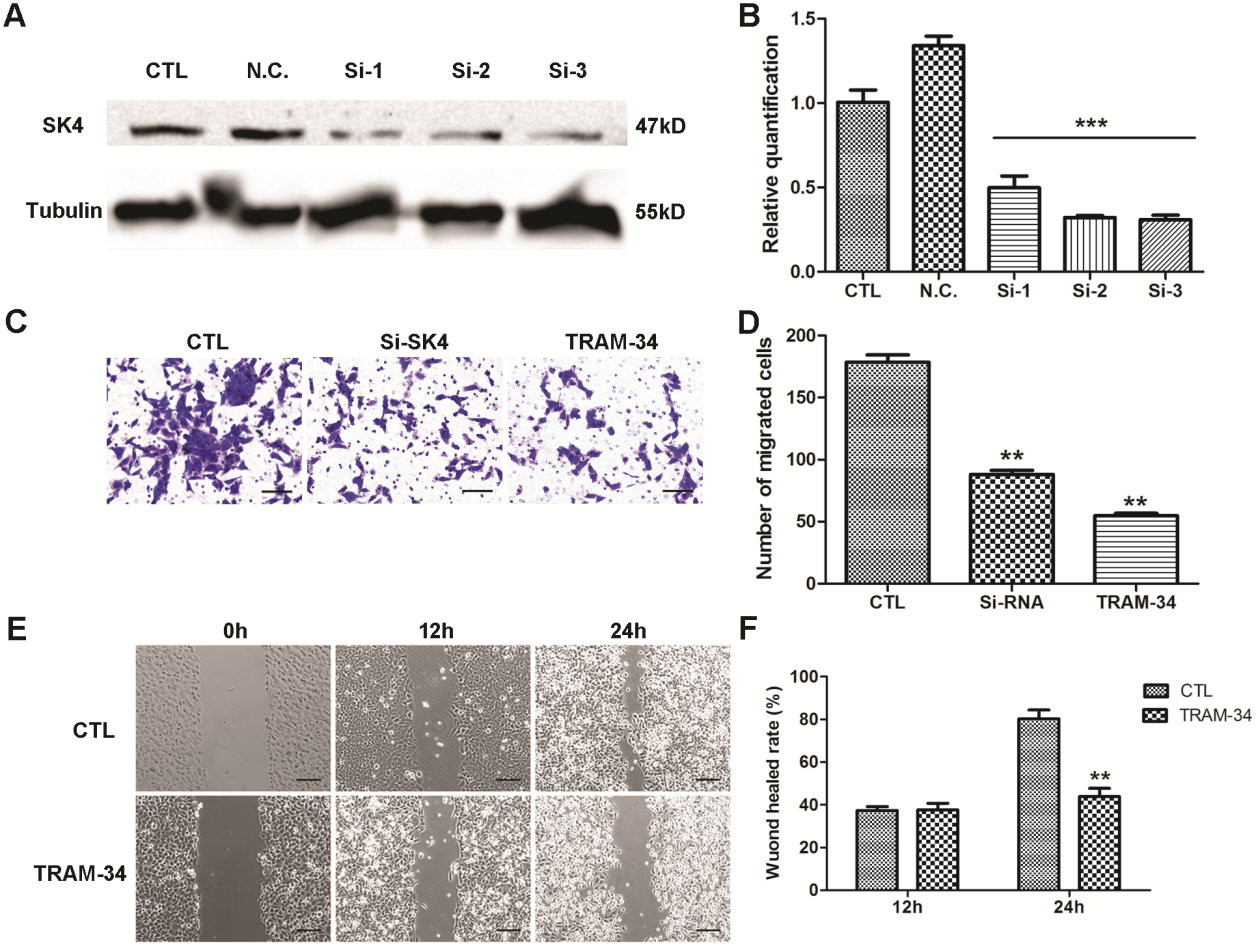
Down-regulation of SK4 channels inhibits the migration of MDA-MB-231 cells. A negative control siRNA (N.C.) and 3 SK4-specific siRNAs (Si-1, Si-2 and Si-3) were transfected into MDA-MB-231 cells, and 20 μM TRAM-34 was added to the TRAM-34-treated group to inhibit SK4 channels. (A, B) Knockdown of SK4 by siRNA was confirmed using immunoblotting and real-time PCR; n = 3. (C, D) The images and bar of the transwell migration assay indicate that the counts of migrated cells in SK4 siRNA (Si-SK4)- and TRAM-34-treated group were significantly less than those of the control (CTL). Scale bars, 50 μm; n = 4. (E, F) The images and bar of the wound-healing assay. The wound-healing rate represents the distance migrated by cells at certain time divided by the wound distance at 0 h. Scale bars, 100 μm; n = 3. The data are presented as the mean ± SD, Dunnett’s Multiple Comparison Test was applied in (B) and (D), and unpaired *t* test in (F). **p<0.01, ***p<0.001.

To suppress the expression of SK4 proteins, a SK4-specific siRNA was used. MDA-MB-231 cells were transfected with SK4 siRNA, and the consequent silencing was confirmed using WB and real-time PCR (Fig 5A and 5B). Next we evaluated the migration ability of MDA-231 cells. During a transwell migration assay, the number of migrated cells in the SK4 siRNA- and TRAM-34-treated groups was 88 ± 7.0 and 55 ± 4.9, respectively, while in the control group, the number increased to 178 ± 11.7 (Fig 5C and 5D). In addition, TRAM-34 was applied in a wound-healing assay, and 24 h after treatment, the wound-healing rate of the 10-μM TRAM-34-treated group was 43.9% ± 5.4, which was substantially lower than that of the control group (80.3% ± 5.9; Fig 5E and 5F). These data suggested that down-regulation of SK4 channels significantly inhibited the migration ability of MDA-MB-231 cells (**p<0.01).

### The EGF/bFGF-induced EMT of MDA-MB-231 cells correlates with SK4 channels

**Fig 6:**
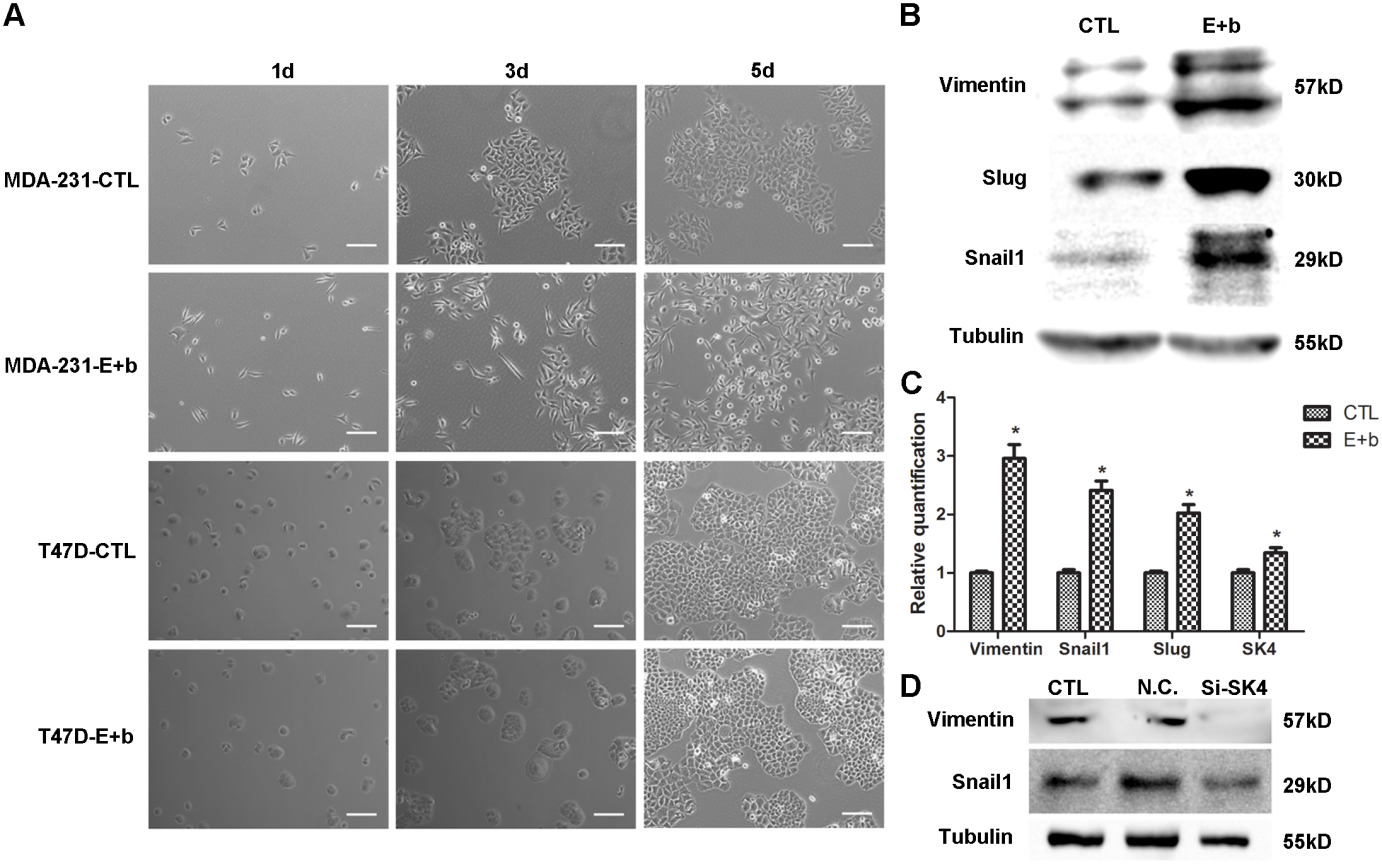
The EGF/bFGF-induced EMT of MDA-MB-231 cells correlates with SK4 channels. (A) Phase contrast images of MDA-231 and T47D cells treated with (E+b) or without (CTL) EGF/bFGF for 1 day, 3 days and 5 days. Scale bars, 100 μm. (B, C) The EGF/bFGF-induced EMT of MDA-231 cells was confirmed using immunoblotting and real-time PCR of EMT markers (Vimentin, Snail1 and Slug), and the SK4 mRNA level increased after EMT. (D) Immunoblotting of EMT-related proteins (Vimentin and Snail1) was performed 72 h after MDA-231 cells were transfected with negative control siRNA (N.C.) or SK4-specific siRNA (Si-SK4); cells that did not undergo transfection served as a control (CTL). The data are presented as the mean ± SD, and paired *t* test was applied in comparison. n = 3; *p<0.05.

Next, we investigated the effect of SK4 channels on the EMT of MDA-MB-231 cells. Firstly, to construct a breast cancer EMT cell model, we applied TGF-β1 and EGF/bFGF to induce EMT and found that none of the breast cancer cell lines could undergo a TGF-β1 (5–10 ng/ml)-induced EMT. However, EGF/bFGF could mediate the EMT of MDA-MB-231 and MDA-MB-468 cells. After treatment with EGF (20 ng/ml) and bFGF (10 ng/ml), MDA-231 cells became elongated with enhanced Vimentin protein and mRNA expression, and up-regulation of the transcription factors (TFs) Slug and Snail1 was observed (Fig 6A–6C). However, with the same treatment, T47D cells did not show a change in their cell morphology (Fig 6A), and neither did MCF-7 cells (S1 Fig). When MDA-231 cells underwent EMT, SK4 mRNA expression was increased (Fig 6C). In addition, down-regulation of SK4 expression suppressed Vimentin and Snail1 expression (Fig 6D). These results revealed that the EGF/bFGF-induced EMT of MDA-231 cells correlated with the expression of SK4 channels.

## Discussion

SK4, also known as KCa3.1 or hIKCa1, encoded by the gene KCNN4, is one member of the calcium-activated K^+^ channels that open in response to cytosolic calcium. This channel mediates a link between cytosolic calcium and membrane potential [13–17]. SK4 channels are widely expressed in epithelial tissues, fibroblasts and hematopoietic-derived cells [18–22]. SK4 channels have physiological effects on fluid secretion, cell volume regulation, cell proliferation and migration.

Moreover, increasing evidence, including our previous research, indicates that SK4 channels participate in the tumor progression. SK4 mRNA is overexpressed in several human cancer cell lines and tissues, such as glioblastoma cells [23], pancreatic cancer cells [24], prostate cancer cells [25], melanoma cells [26], endometrial cancer cells [6] and liver cancer tissue [7]. Recent *in vitro* studies indicate that specific blockers of SK4 channels such as TRAM-34 and clotrimazole can inhibit the proliferation of these cancer cells, further indicating that SK4 channels play an important role in cancer cell proliferation [24, 26]. SK4 channels are also closely related to cancer cell apoptosis, migration and the EMT process [8–10, 27]. However, research on SK4 in TNBC is limited, prompting us to investigate whether SK4 channels are involved in the biologically malignant behavior of TNBC.

In the present study, we explored the expression of SK4 proteins in various subtypes of breast cancer tissues and cell lines. By employing IHC and WB, we demonstrated that increased levels of SK4 protein were present in breast cancer tissues, as is the case for several other tumor types. However, across different subtypes of breast cancer, including TNBC, luminal and HER2 breast cancer, the SK4 protein was strongly expressed with no apparent differences. Interestingly, though the difference in expression level was not significant, concentrated SK4 proteins were observed at the cytomembrane of the TNBC cells. In consideration of the functions of the SK4 channels in cell proliferation, transition and migration, this phenomenon may explain why TNBC relapses early and easily metastasizes.

Regarding the expression of SK4 mRNA and proteins in 4 different breast cancer cell lines, including two TNBC cell lines (MDA-MB-231 and MDA-MB-468), and two luminal breast cancer cell lines (MCF-7 and T47D) [28], we found relatively higher expression levels of SK4 mRNA and protein in the TNBC cell lines and reduced expression in T47D cells. SK4 proteins could be detected on the cell membrane using immunofluorescence. Moreover, during the patch-clamp experiments, we applied 350 nM free Ca^2+^ in the intracellular fluid and found apparent outward currents. The currents were apparently decreased after the TRAM-34 treatment. When no free Ca^2+^ was applied in the pipette solution, the reversal potential turned to the right with diminished TRAM-34 block effect. This result suggests that calcium-activated channels were functional in MDA-MB-231 cells and they could be inhibited by SK4-specific blocker TRAM-34. All of these data indicate that SK4 channels were functionally expressed in TNBC cells.

EMT, the first step towards metastasis, is a transition process in which epithelial cells acquire mesenchymal characteristics. The hallmarks of the EMT can be summarized as the loss of epithelial cells markers such as E-cadherin, the loss of apico-basal polarity, the acquisition of mesenchymal cells markers such as Vimentin, and the acquisition of migratory and invasive properties [29, 30]. An increasing number of studies have indicated that EMT is involved not only in metastatic events, but also in other events such as the resistance to cell death, chemotherapy and immunotherapy [31]. In the present study, we evaluated ER expression and the expression of the EMT-related markers E-cadherin and Vimentin in several cancer cell lines and tissues, confirming that T47D cells were epithelial luminal breast cancer cells and MDA-MB-231 cells were mesenchymal-like TNBC cells. This result suggests that epithelial and mesenchymal cells can be regarded as two extremes of a transition [32]. Besides, the intermediate phenotype of the transition corresponds to a partial EMT that is also observed in certain pathological conditions such as carcinosarcoma, renal and liver epithelial fibrosis [33–35]. Thus, MCF-7, T47D and MDA-468 cells are in the “epithelial” state with relatively higher polarity and lower motility, and MDA-231 cells are in the “mesenchymal” state, with less polarity and greater motility.

Next, we evaluated the effects of SK4 channels on breast cancer cell proliferation and apoptosis. We found that blockage of SK4 channels inhibited cell proliferation and promoted the apoptosis of SK4-positive TNBC cells MDA-MB-231. The results are in agreement with the work of Ouadid-Ahidouch *et al*., who reported that the block of SK4 channels in MCF-7 inhibited cell cycle progression [36]. Interestingly, despite expressing little SK4 protein, T47D cell proliferation was significantly inhibited by clotrimazole and weakly promoted by TRAM-34. This observation can be explained by the fact that clotrimazole inhibits human cytochrome P450 enzymes [37–39], while TRAM-34 selectively inhibits SK4 without blocking cytochrome P450 enzymes [40]. Moreover, another study demonstrated that TRAM-34 stimulated the proliferation of breast cancer cells via the activation of ERs [41]; thus, TRAM-34 may stimulate the growth of ER-positive T47D cells. Therefore, in our study, it is reasonable that TRAM-34 could inhibit cell proliferation and promote the apoptosis of the ER-negative MDA-MB-231 cells.

Given the evidence that calcium-activated potassium channels play an important role in tumor cell motility [9, 42], we investigated the effect of down-regulating SK4 channels on TNBC cell movement. Using SK4-specific siRNA, SK4 coding gene KCNN4 was successfully knocked down, and the motility of the siRNA-SK4 MDA-MB-231 cells was apparently weakened. The blockage of SK4 channels by TRAM-34 had a similar effect. Thus, SK4 may contribute to TNBC cell migration.

Although the half maximal inhibitory concentration (IC50) of TRAM-34 measured by patch-clamp technique is 20 nM [40], high concentration of TRAM-34 was applied *in vitro* or/and *in vivo* studies to generate effective effects. For instance, in one study, 30 μM TRAM-34 was employed to suppress the growth of human endometrial cancer cells and inhibit the progression of human endometrial carcinoma in nude mice [6]. Another study proved that migration-associated secretion of melanoma is diminished by TRAM-34 at a concentration of 40 μM [27]. During the investigation the effects of SK4 channels on breast cancer cell growth and migration, we found that the effective concentration of TRAM-34 was greater than 8 μM.

Finally, based on our founding that SK4 is up-regulated and E-cadherin is down-regulated in breast cancer tissues, we explored whether SK4 channels participated in the EMT progress of breast cancer cells. As TGF-β1-mediated induction of an EMT is a rare event *in vitro* [43], and we confirmed that the commonly used cell factor TGF-β1 could not induce EMT in the four breast cancer cell lines. By employing another routine method [31], MDA-MB-231 and MDA-MB-468 cells, but not MCF-7 and T47D cells, underwent an EMT that was successfully mediated by the cell growth factor EGF combined with bFGF. Approximately 3 days after treatment with EGF/bFGF, MDA-MB-231 cells were spindle-like and exhibited up-regulated levels of Vimentin and EMT-related transcription factors. One explanation for these results is that several breast cancer cell lines are insensitive to TGF-β1 with respect to the EMT; in contrast, various breast cancer cell lines may in different EMT stages, and TNBC cell lines may more easily adopt a mesenchymal phenotype. In addition, after the EMT of MDA-231, enhanced SK4 mRNA expression was detected. To further confirm the relationship between SK4 channels and EMT, we down-regulated SK4 expression and found that Vimentin and Snail1 expression was also suppressed. These results indicated that the EMT process of MDA-MB-231 cells correlated with the expression of SK4 channels.

In summary, our results provide evidence that SK4 channels are expressed in TNBC and that inhibition of these channels suppresses the proliferation, migration and EMT process of TNBC cells. SK4 may be a potential and novel target for TNBC treatment.

## Supporting Information

S1 Fig MDA-MB-468 and MCF-7 cells treated with EGF/bFGF. Phase contrast images of MDA-468 and MCF-7 cells treated with (E+b) or without (CTL) EGF/bFGF for 1 day, 3 days and 5 days. Scale bars, 100 μm.
(TIF)

S1 File Data statement. Related data in Figs 1–6 and Table 1 were stated separately.
(ZIP)

S1 Table Primer sequences of relevant genes. (DOCX)

S2 Table siRNA sequences and target sequences. (DOCX)
