# Supplementary figures and images for: Inhibition of SK4 Potassium Channels Suppresses Cell Proliferation, Migration and the Epithelial-Mesenchymal Transition in Triple-Negative Breast Cancer Cells
Source: PLoS One. 2016 Apr 28;11(4):e0154471. doi: 10.1371/journal.pone.0154471 (PMC4849628; doi:10.1371/journal.pone.0154471)

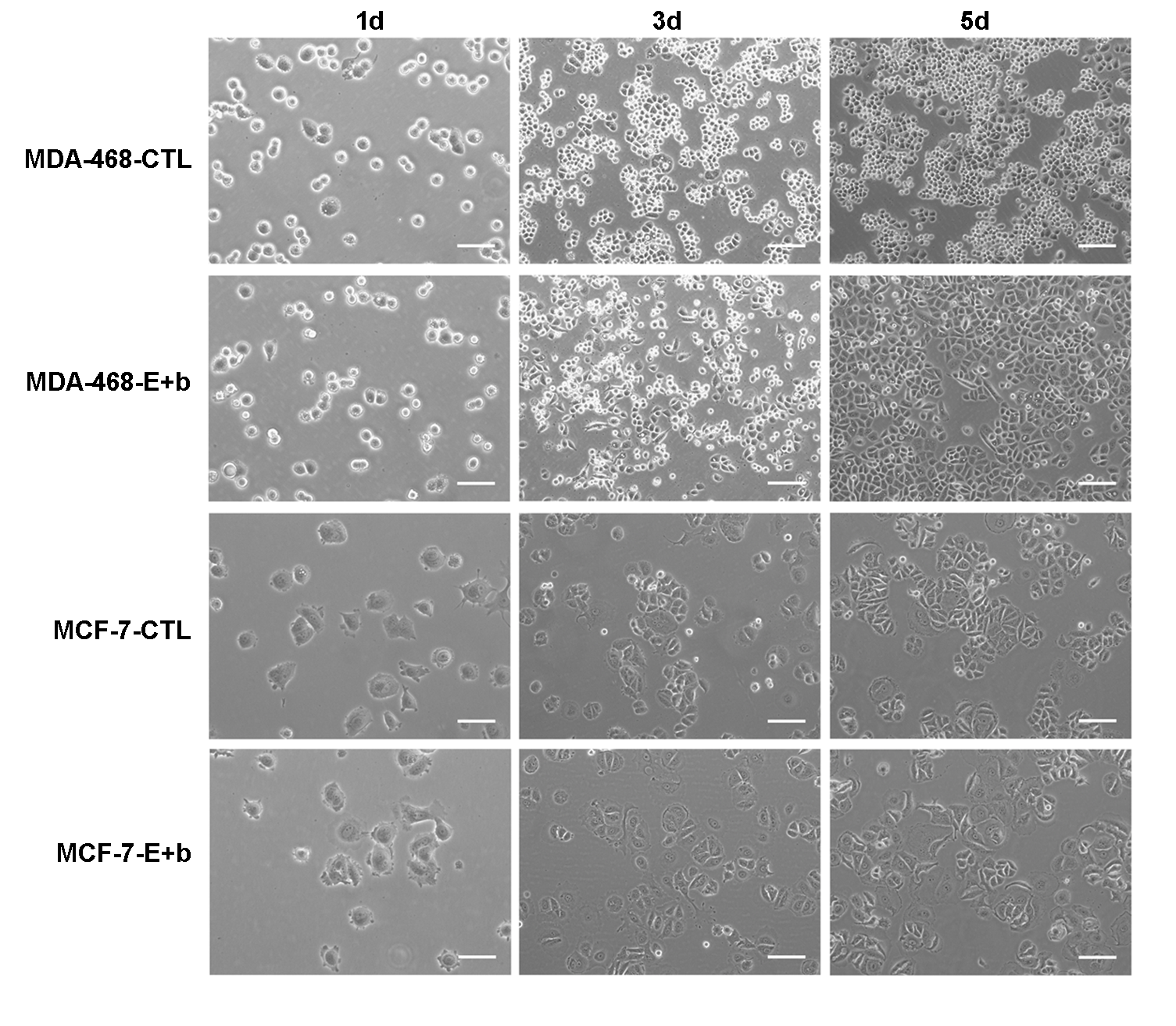

Supplement: S1 Fig — Phase contrast images of MDA-468 and MCF-7 cells treated with (E+b) or without (CTL) EGF/bFGF for 1 day, 3 days and 5 days. Scale bars, 100 μm. (TIF) [file pone.0154471.s001.tif]
