# Supplementary material for: Inhibition of SK4 Potassium Channels Suppresses Cell Proliferation, Migration and the Epithelial-Mesenchymal Transition in Triple-Negative Breast Cancer Cells
Source: PLoS One. 2016 Apr 28;11(4):e0154471. doi: 10.1371/journal.pone.0154471 (PMC4849628; doi:10.1371/journal.pone.0154471)
Supplement: S1 Table — (DOCX) [file pone.0154471.s003.docx]

**S1 Table.**

| Gene | GenBank Number | Primer Sequence |
| --- | --- | --- |
| KCNN4 | [NM_002250.2](http://www.ncbi.nlm.nih.gov/entrez/viewer.fcgi?db=nucleotide&id=25777651) | Forward: GCAGGTGGATCTATTTCA  Reverse: TCGTATCACAGCAGGTTA |
| E-cadherin | [NM_004360.3](http://www.ncbi.nlm.nih.gov/entrez/viewer.fcgi?db=nucleotide&id=169790842) | Forward: CTTTGACGCCGAGAGCTAC  Reverse: TTTGAATCGGGTGTCGAGGG |
| Vimentin | [NM_003380.3](http://www.ncbi.nlm.nih.gov/entrez/viewer.fcgi?db=nucleotide&id=240849334) | Forward: TCAGAATATGAAGGAGGAAATGGC  Reverse: GAGTGGGTATCAACCAGAGGGAGT |
| Snail1 | NM_005985.3 | Forward: ATTTCAGCCTCCTGTTTGGT  Reverse: AAGTGACAGCCATTACTCA |
| Snail2/Slug | NM_003068.4 | Forward: ACAGCGAACTGGACACACAT  Reverse: GAGAGAGGCCATTGGGTAGC |
| GAPDH | NM_002046 | Forward: AATCCCATCACCATCTTCCAG  Reverse: GAGCCCCAGCCTTCTCCAT |
