# Supplementary material for: Inhibition of SK4 Potassium Channels Suppresses Cell Proliferation, Migration and the Epithelial-Mesenchymal Transition in Triple-Negative Breast Cancer Cells
Source: PLoS One. 2016 Apr 28;11(4):e0154471. doi: 10.1371/journal.pone.0154471 (PMC4849628; doi:10.1371/journal.pone.0154471)
Supplement: S2 Table — (DOCX) [file pone.0154471.s004.docx]

**S2 Table.**

| siRNA | Target Sequence | siRNA Sequence (5’-3’) |
| --- | --- | --- |
| Si-1 | CATCGGCGCTCTCAATCAA | Sense: GCACCUUUCAGACACACUU  Anti-sense: AAGUGUGUCUGAAAGGUGC |
| Si-2 | CCTGTTCCTGGTTAAATGC | Sense: CCUGUUCCUGGUUAAAUGC  Anti-sense: GCAUUUAACCAGGAACAGG |
| Si-3 | CATCGGCGCTCTCAATCAA | Sense: CAUCGGCGCUCUCAAUCAA  Anti-sense: UUGAUUGAGAGCGCCGAUG |
